# Supplementary material for: Single cell spatial transcriptomics integration deciphers the morphological heterogeneity of atherosclerotic carotid arteries
Source: Nat Commun. 2025 Dec 18;16:11282. doi: 10.1038/s41467-025-67679-4 (PMC12717225; doi:10.1038/s41467-025-67679-4)
Supplement: Supplementary file 2 — Description Of Additional Supplementary File [file 41467_2025_67679_MOESM2_ESM.pdf]

Description of Additional supplementary files

Supplementary Data1: showing the individual genes selected for each gene panel used for spatial transcriptomic sequencing.

Supplementary Data2: showing the differential gene expression (DGE) results across conditions levels with Xenium Panel 1 genes. DGE was performed within low-level cell type clusters. The sheet names depict the DGE method used and condition whose levels were compared.

Supplementary Data3: showing the differential gene expression (DGE) results across conditions levels with Xenium Panel 2 genes. DGE was performed within low-level cell type clusters. The sheet names depict the DGE method used and condition whose levels were compared.

Supplementary Data4: showing the differential gene expression (DGE) results across 'Smoking ever'conditions levels of bulk RNA-seq data. DGE was performed within plaque samples. The sheet names depict the DGE method used and condition whose levels were compared.

Supplementary Data5: showing the differential gene expression (DGE) results across 'Smoking ever'conditions levels of bulk RNA-seq data, with overlapping genes of the same comparison in Xenium. DGE was performed within plaque samples. The sheet names depict the DGE method used and condition whose levels were compared.

Supplementary Data6: showing the differential gene expression (DGE) results across high-level cell types of our scRNA-seq data. The sheet names depict the DGE method.

Supplementary Data7: showing the differential gene expression (DGE) results across low-level cell types of our scRNA-seq data. DGE was performed within high-level cell type clusters.The sheet names depict the DGE method.

Supplementary Data8: showing the differential gene expression (DGE) results across conditions levels with our scRNA-seq data. DGE was performed within low-level cell type clusters. The sheet names depict the DGE method used and condition whose levels were compared.

Supplementary Data9: showing the differential gene expression (DGE) results across high-level cell types of Xenium Panel 1. The sheet names depict the DGE method and Baysor scale parameter used for segmentation.

Supplementary Data10: showing the differential gene expression (DGE) results across high-level cell types of Xenium Panel 2. The sheet names depict the DGE method and Baysor scale parameter used for segmentation.

Supplementary Data11: showing the differential gene expression (DGE) results across low-level cell types of Xenium Panel 1. DGE was performed within high-level cell type clusters.The sheet names depict the DGE method and Baysor scale parameter used for segmentation.

Supplementary Data12: showing the differential gene expression (DGE) results across low-level cell types of Xenium Panel 2. DGE was performed within high-level cell type clusters.The sheet names depict the DGE method and Baysor scale parameter used for segmentation.

Supplementary Data13: showing the differential gene expression (DGE) results across conditions levels with Xenium Panel 1 genes. DGE was performed within low-level cell type clusters of media cells of patients 1-4. The sheet names depict the DGE method used and condition whose levels were compared.

Supplementary Data14: showing the differential gene expression (DGE) results across conditions levels with Xenium Panel 2 genes. DGE was performed within low-level cell type clusters of media cells of patients 1-4. The sheet names depict the DGE method used and condition whose levels were compared.

Supplementary Data15: showing permutation test results of Panel 1& 2 neighbourhood clusters, using neighbourhood enrichment scores as metric.

Supplementary Data16: showing the differential gene expression (DGE) results across macrophage substates of plaques 4,7,8,10 with Xenium Panel 1 genes.

Supplementary Data 17: Raw count matrix of bulk RNA-seq data

Supplementary Data18: showing the metadata of patients used in the bulk RNA-seq dataset.
