# Supplementary material for: Single cell spatial transcriptomics integration deciphers the morphological heterogeneity of atherosclerotic carotid arteries
Source: Nat Commun. 2025 Dec 18;16:11282. doi: 10.1038/s41467-025-67679-4 (PMC12717225; doi:10.1038/s41467-025-67679-4)
Supplement: Supplementary file 4 — Reporting Summary [file 41467_2025_67679_MOESM4_ESM.pdf]

nature portfolio

Corresponding author(s): Lars Maegdefessel, Michael P. Menden

Last updated by author(s): Nov 2, 2025

Reporting Summary

Nature Portfolio wishes to improve the reproducibility of the work that we publish. This form provides structure for consistency and transparency in reporting. For further information on Nature Portfolio policies, see our Editorial Policies and the Editorial Policy Checklist.

Statistics

For all statistical analyses, confirm that the following items are present in the figure legend, table legend, main text, or Methods section.

n/a	Confirmed	
☐	☒	The exact sample size (*n*) for each experimental group/condition, given as a discrete number and unit of measurement
☐	☒	A statement on whether measurements were taken from distinct samples or whether the same sample was measured repeatedly
☐	☒	The statistical test(s) used AND whether they are one- or two-sided<br>*Only common tests should be described solely by name; describe more complex techniques in the Methods section.*
☐	☒	A description of all covariates tested
☐	☒	A description of any assumptions or corrections, such as tests of normality and adjustment for multiple comparisons
☐	☒	A full description of the statistical parameters including central tendency (e.g. means) or other basic estimates (e.g. regression coefficient) AND variation (e.g. standard deviation) or associated estimates of uncertainty (e.g. confidence intervals)
☐	☒	For null hypothesis testing, the test statistic (e.g. *F*, *t*, *r*) with confidence intervals, effect sizes, degrees of freedom and *P* value noted<br>*Give P values as exact values whenever suitable.*
☒	☐	For Bayesian analysis, information on the choice of priors and Markov chain Monte Carlo settings
☒	☐	For hierarchical and complex designs, identification of the appropriate level for tests and full reporting of outcomes
☐	☒	Estimates of effect sizes (e.g. Cohen's *d*, Pearson's *r*), indicating how they were calculated

Our web collection on statistics for biologists contains articles on many of the points above.

Software and code

Policy information about availability of computer code

Data collection	No software was used
Data analysis	Data analysis was performed with Python (3.9.17 and 3.10), R (4.1.2) and RStudio (4.2.1)

For manuscripts utilizing custom algorithms or software that are central to the research but not yet described in published literature, software must be made available to editors and reviewers. We strongly encourage code deposition in a community repository (e.g. GitHub). See the Nature Portfolio guidelines for submitting code & software for further information.

Data

Policy information about availability of data

All manuscripts must include a data availability statement. This statement should provide the following information, where applicable:

- Accession codes, unique identifiers, or web links for publicly available datasets
- A description of any restrictions on data availability
- For clinical datasets or third party data, please ensure that the statement adheres to our policy

Data from our own scRNA-Seq and single cell spatial transcriptomics experiments have been deposited in NCBI's Gene Expression Omnibus. The previously generated datasets, that are already published, are accessible through GEO Series accession number GSE247238 (scRNA-Seq, patients 1-10) or in this manuscript in Suppl. Table 6 and Suppl. Table 7 (bulk seq data). Newly acquired datasets for this study are: GSE294291 (scRNA-Seq, patients 11-17) and GSE294466 (Xenium 2nature portfolio | reporting summary April 2023

spatial transcriptomics). An additional external scRNA-Seq dataset of human carotid plaques using a similar set-up of plaque vs. adjacent control was downloaded from the GEO repository (GSE159677).

Research involving human participants, their data, or biological material

Policy information about studies with human participants or human data. See also policy information about sex, gender (identity/presentation), and sexual orientation and race, ethnicity and racism.

Reporting on sex and gender	Sex was considered, but not in the study design. After selecting available samples of patients, we later declared and added sex as a clinical variable. Sex of participants was determined based on self-report.
Reporting on race, ethnicity, or other socially relevant groupings	Race, ethnicity, or other socially relevant groupings were not assessed in this study.
Population characteristics	See above
Recruitment	Patients come to our clinic (Vascular and Endovascular Surgery at TUM University Hospital in Munich) to undergo carotid endarterectomy. Apart from a filled in consent and the present of the tissue, no other criteria had to be met in order to be recruited.
Ethics oversight	Human samples were obtained from patients diagnosed with carotid stenosis and sourced from the Munich Vascular Biobank. The collection process adhered to the ethical principles of the Declaration of Helsinki and was approved by the local ethics committee at the Technical University of Munich (approval numbers: 2799/10 and 2023-297). Informed written consent was obtained from all participants, and clinical data were retrieved from electronic patient records.

Note that full information on the approval of the study protocol must also be provided in the manuscript.

Field-specific reporting

Please select the one below that is the best fit for your research. If you are not sure, read the appropriate sections before making your selection.

☒ Life sciences ☐ Behavioural & social sciences ☐ Ecological, evolutionary & environmental sciences

For a reference copy of the document with all sections, see nature.com/documents/nr-reporting-summary-flat.pdf

Life sciences study design

All studies must disclose on these points even when the disclosure is negative.

Sample size	e In this study we included all available samples at this moment: n=297 bulk RNA seq samples, n=17 single cell seq patients (n=33 samples) and<br>n=12 Xenium patients (n=16 samples)
Data exclusions	No data was excluded.
Replication	No replication was performed.
Randomization	No randomization was performed.
Blinding	No blinding was performed.

Reporting for specific materials, systems and methods

We require information from authors about some types of materials, experimental systems and methods used in many studies. Here, indicate whether each material, system or method listed is relevant to your study. If you are not sure if a list item applies to your research, read the appropriate section before selecting a response.

Materials & experimental systems

n/a | Involved in the study
- ☒ ☐ Antibodies
- ☒ ☐ Eukaryotic cell lines
- ☒ ☐ Palaeontology and archaeology
- ☒ ☐ Animals and other organisms
- ☒ ☐ Clinical data
- ☒ ☐ Dual use research of concern
- ☒ ☐ Plants

Methods

n/a | Involved in the study
- ☒ ☐ ChIP-seq
- ☒ ☐ Flow cytometry
- ☒ ☐ MRI-based neuroimaging

Plants

Seed stocks	*Report on the source of all seed stocks or other plant material used. If applicable, state the seed stock centre and catalogue number. If plant specimens were collected from the field, describe the collection location, date and sampling procedures.*
Novel plant genotypes	*Describe the methods by which all novel plant genotypes were produced. This includes those generated by transgenic approaches, gene editing, chemical/radiation-based mutagenesis and hybridization. For transgenic lines, describe the transformation method, the number of independent lines analyzed and the generation upon which experiments were performed. For gene-edited lines, describe the editor used, the endogenous sequence targeted for editing, the targeting guide RNA sequence (if applicable) and how the editor was applied.*
Authentication	*Describe any authentication procedures for each seed stock used or novel genotype generated. Describe any experiments used to assess the effect of a mutation and, where applicable, how potential secondary effects (e.g. second site T-DNA insertions, mosiacism, off-target gene editing) were examined.*
